# *Cleistocalyx nervosum* var. *paniala* mitigates oxidative stress and inflammation induced by PM_10_ soluble extract in trophoblast cells via miR-146a-5p

**DOI:** 10.1038/s41598-024-73000-y

**Published:** 2024-10-16

**Authors:** Wittaya Chaiwangyen, Orawan Khantamat, Komsak Pintha, Napapan Kangwan, Amnart Onsa-ard, Piyawan Nuntaboon, Angkana Songkrao, Pilaiporn Thippraphan, Dana Chaiyasit, Francisco Lázaro Pereira de Sousa

**Affiliations:** 1 Division of Biochemistry, School of Medical Sciences, University of Phayao, Phayao, 56000 Thailand; 2 Department of Biochemistry, Faculty of Medicine, Chiang Mai University, Chiang Mai, 50200 Thailand; 3 Division of Physiology, School of Medical Sciences, University of Phayao, Phayao, 56000 Thailand; 4 Clinical Chemistry Laboratory, Chiang Rai Prachanukroh Hospital, Chiang Rai, 57000 Thailand; 5 Department of Gynecology and Obstetrics, UNILUS (Centro Universitário Lusíada), Santos, 11050-071 Brazil

**Keywords:** PM_10_, *Cleistocalyx nervosum* var. *paniala*, MiR-146a-5p, Trophoblast, Pregnancy, Oxidative stress, SOX5, Environmental sciences, Cell biology

## Abstract

Air pollution poses a significant global concern, notably impacting pregnancy outcomes through mechanisms such as DNA damage, oxidative stress, inflammation, and altered miRNA expression, all of which can adversely affect trophoblast functions. *Cleistocalyx nervosum* var. *paniala*, known for its abundance of anthocyanins with diverse biological activities including anti-mutagenic, antioxidant, and anti-inflammatory properties, is the focus of this study examining its effect on Particulate Matter 10 (PM_10_) soluble extract-induced trophoblast cell dysfunction via miRNA expression. The study involved the extraction of *C. nervosum* fruit using 70% ethanol, followed by fractionation with hexane, dichloromethane, and ethyl acetate. Subsequent testing for total phenolics, flavonoids, anthocyanins, and antioxidant activity revealed the ethyl acetate fraction (CN-EtOAcF) as possessing the highest phenolic and anthocyanin content along with potent antioxidant activity, prompting its selection for further investigation. In vitro studies on HTR-8/SVneo cells demonstrated that 5–10 µg/mL PM_10_ soluble extract exposure inhibited cell proliferation, migration, invasion, and induced apoptosis. However, pretreatment with 20–80 µg/mL CN-EtOAcF followed by 5 µg/mL PM_10_ soluble extract exposure exhibited protective effects against PM_10_ soluble extract-induced damage, including inflammation inhibition and intracellular ROS suppression. Notably, CN-EtOAcF down-regulated PM_10_-induced miR-146a-5p expression, with SOX5 identified as a potential target. Overall, CN-EtOAcF demonstrated the potential to protect against PM_10_-induced harm in trophoblast cells, suggesting its possible application in future therapeutic approaches.

**Supplementary Information:**

The online version contains supplementary material available at 10.1038/s41598-024-73000-y.

## Introduction

During the implantation process, the effective penetration of the maternal decidua and myometrium by extravillous trophoblast (EVT) is vital for a successful pregnancy as it facilitates the modification of maternal spiral arteries. This modification results in the widening of these arteries, enhancing the provision of nutrients and facilitating gas exchange for fetal development. There have been reports of a connection between deficient trophoblast invasion and pregnancy complications such as preterm birth, preeclampsia, fetal growth restriction, and spontaneous abortion. The defected trophoblast functions and pregnancy disorders are also associated with environmental pollutants, especially particulate matter (PM) air pollution^1,2^.

Environmental air pollution, specifically fine PM with diameter < 2.5 μm (PM_2.5_) and coarse PM with diameter < 10 μm (PM_10_), is recognized as having significant adverse effects on maternal and neonatal health^3^. Several studies have revealed that PM_2.5_ and PM_10_ have the capability to breach the lung barrier and access the blood circulation^4^, triggering oxidative stress and inflammation, which in turn elicit systemic responses and contribute to various diseases^5,6^. PM_2.5_ particles can penetrate deeply into the respiratory system and enter the bloodstream, affecting various organs including the placenta, indicating systemic exposure^7–11^. Conversely, the systemic translocation of PM_10_, typically remain in the upper respiratory tract^12^, is less well documented. Studies have shown that both PM_2.5_ and PM_10_ soluble extracts contain similar chemical constituents, including elements and polycyclic aromatic hydrocarbons (PAHs)^13–15^. This chemical similarity justifies the use of PM_10_ soluble extracts in the study, which aims to explore trophoblast cell functions through miR-146a-5p expression associated with PM_10_.

In animal models, exposure to PM_2.5_ has been shown to decrease fetal weight and crown-rump length in mice, along with impairments in placental trophoblast syncytialization^16^. Airborne PM_2.5_ exposure has also been linked to disruptions in trophoblast cellular processes, resulting in growth inhibition, inflammation, endoplasmic reticulum stress, and impaired migration and invasion abilities^2,17^, potentially leading to pregnancy complications. Previous research conducted by our team revealed that exposure to PM_10_ suppressed the proliferation, migration, and invasion of trophoblast HTR-8/SVneo cells. Furthermore, PM_10_ exposure induced inflammation in trophoblast cells by upregulating the expression levels of proinflammatory cytokines such as IL-1β, IL-6, and TNF-α^18^.

Lately, an increasing number of studies have been investigating the relationship between exposure to PM and the expression of miRNAs. This interest stems from the fact that miRNAs have a substantial impact on a range of cellular processes such as cell differentiation, proliferation, migration, invasion, death, and development^19^. miRNA, a small non-coding RNA consisting of 21–23 nucleotides, specifically binds to sequences within the 3’ UTR of its target mRNAs, resulting in the suppression of gene expression either by inhibiting translation or promoting mRNA degradation^20^. Throughout pregnancy, miRNAs oversee the regulation of trophoblast cell functions, and any disruption in miRNA expression can result in trophoblast dysfunction, potentially leading to pregnancy complications^21–23^. Numerous circulating miRNAs were differentially expressed upon exposure to PM including miR-146a-5p^24–26^.

miR-146a-5p is recognized as a tumor suppressor in various types of cancer, with the ability to inhibit cancer cell proliferation, migration, invasion, and epithelial-mesenchymal transition by targeting SOX5 ^27^. Furthermore, miR-146a-5p plays a crucial role in modulating the inflammatory response^28^. An increase in miR-146a-5p expression in preeclampsia placenta has been documented, with its role in inhibiting trophoblast cell invasion, proliferation, and migration through the regulation of Wnt2 expression^29^. It has been reported that exosomal miR-146a-5p originating from human umbilical cord mesenchymal stem cells can mitigate trophoblast injury and placental dysfunction. This occurs through the regulation of the TRAF6/NF-κB axis, leading to the suppression of IL-1β and IL-18, thereby reducing inflammation^30^. Despite the insights provided by these studies, the relationship between miR-146a-5p expression in trophoblast cells remains unclear.

*Cleistocalyx nervosum* var. *paniala* (Ma Kiang), a member of the Myrtaceae family, is a native berry of Thailand. Its dark purple ripe fruit has been shown to contain a significant amount of anthocyanins and to possess various biological activities, including anti-mutagenic, anti-aging, anti-carcinogenic, antioxidant, and anti-inflammatory properties^31–33^. The *C. nervosum* fruit extract (CNE) has been found to notably inhibit IL-1β-induced inflammation through the inhibition of the production of inflammatory molecules, such as IL-6, MCP-1, and IL-8, in human retinal pigment epithelial cells^33^. Given the biological properties of *C. nervosum* fruit and the association between stress-inducing agent, PM_10_ soluble extract exposure and miR-146a-5p expression in trophoblast cells as discussed above, our objective is to explore the protective properties of CNE against PM_10_ soluble extract-induced oxidative stress, inflammation, and trophoblast cell dysfunction by assessing miR-146a-5p expression.

## Materials and methods

### Plant material collection

*Cleistocalyx nervosum* fruits were collected in August 2022 in Phayao province, Thailand (a latitude of 19° 04′ 21.68′ N and a longitude of 99° 53′ 3.58′ E). A voucher specimen (Code: QBG No. 14630-32) of the plant was acquired and deposited at the Herbarium of the Queen Sirikit Botanic Garden in Chiang Mai, Thailand. The ripe fruit pulp of *C. nervosum* was separated from the seeds and subjected to a 96 h drying process at 40 °C. The resulting dried fruit pulp was finely ground into a powder and stored at −20 °C for future experiments.

### Extraction of plant material^34^

The preparation of the *C. nervosum* fruit extract (CNE) was prepared as follows: the dried powder was initially extracted using 70% ethanol, and the resulting dried crude extract was then fractionated through hexane, dichloromethane, and ethyl acetate. The solvent was eliminated via a rotary evaporator. The water fraction that remained was obtained through the process of freeze-drying. The extracts obtained were weighed for yield assessment and subsequently stored at -20 °C for future analysis. The percentage yield was calculated using the following formula:

$$\% {\text{ yield}}=\left[ {{\text{Weight of extract }}\left( {\text{g}} \right)/{\text{Weight of dried material }}\left( {\text{g}} \right)} \right] \times {\text{1}}00$$

### Phytochemical determination

#### Total phenolic content (TPC)

The TPC in the extracts was assessed using the Folin–Ciocalteu reagent. Concisely, either diluted samples or a standard (Gallic acid) were combined with a solution of Folin–Ciocalteu and sodium carbonate. After 30 min incubation, the absorbance of the reaction was measured at 765 nm. TPC was quantified in milligrams of gallic acid equivalents per gram of plant dry weight (mg GAE/g extract).

#### Total flavonoid content (TFC)

The TFC in the extracts was assessed through a colorimetric method using aluminum chloride. Briefly, either diluted samples or a standard (Catechin) were combined with a sodium nitrite solution and allowed to incubate for 5 min at room temperature. Following incubation, an aluminum chloride solution and sodium hydroxide were introduced into the mixture. The absorbance of the resulting reaction was detected after a 10 min incubation period at 510 nm. TFC was measured in milligrams of catechin equivalents per gram of plant dry weight (mg CE/ g extract).

### Determination of total anthocyanins by pH differential spectroscopic method^35^

The method for determining total anthocyanin content, as established by Connor et al. in 2002[61]. In this method, each extract was diluted in a solution of 1% HCl in methanol to achieve an absorbance falling within the range of 0.500 to 1.000 at 530 nm. Briefly, 0.25 mL of the extracts were mixed with one mL of two distinct solutions: one with a pH of 1.0 containing 0.025 M potassium chloride, and the other with a pH of 4.5 containing 0.4 M sodium acetate. Following a 30 min incubation at room temperature, absorbance was measured at 520 and 700 nm. The results were presented as milligrams of cyanidin-3-glucoside (c3g) equivalents per 100 g of fresh weight, using a molar extinction coefficient of 27.900. All measurements were performed in triplicate. The results were computed as follows:

$${\text{A}}=({{\text{A}}_{{{520}}}}-{{\text{A}}_{{{700}}}}){\text{pH 10.0}}-({{\text{A}}_{{{520}}}}-{{\text{A}}_{{{700}}}}){\text{ pH 4.5}}$$

The total anthocyanin content was calculated as follows:

$${\text{Total anthocyanin content}}=({\text{A}} \times {\text{MW}} \times {\text{DF}} \times {{1}}000)/({\upvarepsilon} \times {\uplambda} \times {\text{m}}),$$

In this context, MW denotes molecular weight of cyaniding-3-glucoside, ε represents molar extinction coefficient of 27.900, DF represents the dilution factor, λ represents the optical path length of the cuvette (1 cm) and m represents the weight of the sample (in g). The total anthocyanin content was indicated as mg of anthocyanin per g of extract.

### High-performance liquid chromatography (HPLC)

HPLC analysis was carried out using a Shimadzu HPLC system integrated with a UV-VIS diode array detector (LC-20 A, Shimadzu, Japan) for quantification at 280 nm. The column utilizing was a C-18 HPLC column (Allure-C18, Restek, Bellefonte, PA, USA). Maintain column temperature at 30 ºC during analysis. Prepared samples were injected at a volume of 10 µL. Solvent A presented of 0.1%TFA in water, where solvent B comprised 0.1% Trifluoroacetic acid (TFA) in methanol. The solvent gradient applied to all samples and reference standards (Cyanidin-3,5-diglucoside, Cyanidin-3-O-glucoside chloride, Cyanidin-3-O-rutinoside chloride, Pelargonidin-3-O-glucoside chloride, and Peonidin-3-O-glucoside chloride) followed this pattern: 0 to 40 min with a flow rate at 1.0 mL/min. The anthocyanin content was assessed using the external standard method, based on a comparison of their retention times with pure standards^36^.

### Antioxidant determinations

#### DPPH radical scavenging activity

The ability of the CNE to act as hydrogen atoms or electron donors was assessed by observing the lowering of the steady 1,1-diphenyl-2-picryl hydrazyl (DPPH) radical. Various concentration of the CNE or standard Trolox were introduced into a DPPH solution. Following a 30 min incubation period in darkness at room temperature, the absorbance was measured at 515 nm. The percentage of DPPH radical scavenging activity was assessed as the follows:

$${\text{DPPH radical scavenging activity }}\left( \% \right)={\text{ }}[\left( {{{\text{A}}_0} - {{\text{A}}_{\text{s}}}/{{\text{A}}_0}} \right] \times {\text{1}}00$$

where A_0_ represents the absorbance of the control and A_s_ represents the absorbance of the samples. The IC_50_ values were determined by plotting the percentage of inhibition against the concentration of the extracts.

#### ABTS radical scavenging activity

The assessment of free-radical-scavenging activity was conducted using 2,2′- azinobis (3-ethylbenzothiazoline-6-sulfonic acid) ABTS radical cation (ABTS^•+^) decolorization assay (Nile and Park, 2014c)[62]. ABTS^•+^ was generated by mixing 7.5 mM ABTS stock solution with 2.45 mM potassium persulfate, allowing it to remain in the dark at room temperature for 12–16 h before utilization. Prior to the investigation, the ABTS^•+^ mixture was diluted with deionized water to achieve an absorbance value of 0.700 ± 0.02 at 734 nm. The absorbance of the extracts or standard Trolox in the ABTS^•+^ solution was measured at 734 nm after 6 min of addition. The following formula was used to calculate the percentage of ABTS radical scavenging activity:

$${\text{ABTS radical scavenging activity }}\left( \% \right)=\left[ {{\text{1}} - {{\text{A}}_{\text{s}}}/{{\text{A}}_0}} \right] \times {\text{1}}00$$

where A_0_ and A_s_ correspond to the absorbance of control and the samples, respectively. The IC_50_ values were calculated by graphing the inhibition percentage against the extract concentration.

#### Ferric reducing antioxidant power (FRAP) assay

The test was developed to assess the ability of an antioxidant compound to turn ferric ions (Fe^3+^) to ferrous ions (Fe^2+^). This conversion leads to the formation of a blue complex (Fe^2+^/TPTZ). Briefly, the sample and the FRAP reagent were combined, and the absorbance was obtained at 593 nm following a 30 min incubation period. The data was represented in µM equivalents of ferrous ion per gram of the extract^37^.

### PM_10_ sample collection

PM_10_ samples were gathered at the University of Phayao in Phayao, Thailand, during the period of January to March in 2020, which was characterized by a significant occurrence of forest fires. These samples were obtained using 20.3 × 25.4 cm quartz-fiber filters (Toyo Roshi Kaisha, Ltd., Tokyo, Japan) with an Ecotech Model 3000 PM_10_ high-volume air sampler (Ecotech Pty. Ltd., Melbourne, Australia). The filters were collected and stored at −20 °C for further use.

### PM_10_ extraction

The filtered samples were divided into small pieces and separated for 15 min in an ultrasonic bath with a 1:1 combination of hexane and dichloromethane. The resulting substance was then filtered using a 0.45 μm Polytetrafluoroethylene (PTFE) filter for removing insoluble substances. The extracted PM_10_ was subsequently dried with a rotary evaporator before being stored at −20 °C for investigation^18^.

### Particle size and elemental analysis of particulate matter

The size of the particles and chemical content were analyzed at the University of Phayao’s central laboratory in Phayao, Thailand, using scanning electron microscopy (SEM) (FEI Quanta FEG 250, FEI Company, WA, USA) and an integrated energy-dispersive X-ray system (EDX) (Oxford INCA X-Act, Oxford Instruments. Following SEM determination, filter samples measuring 1 mm × 1 mm were obtained from the middle portion of the filter. A gold sputter coater with a vacuum coating system (Quorum SC7620, East Sussex, UK) was used for coating a thin film of gold (Au) to the sample surface. The micrographs of particulate matter were analyzed using xT microscope control software. To determine the precise chemical composition of PM_10_, EDX spectra were acquired, and the weight was determined percentage of every compound contained in the spectrum. Fifteen elements were identified by SEM-EDX, including Oxygen (O), Carbon (C), Silicon (Si), Potassium (K), Aluminum (Al), Zinc (Zn), Sulfur (S), Chloride (Cl), Copper (Cu), Magnesium (Mg), Calcium (Ca), Iron (Fe), Nickel (Ni), Sodium (Na), and Nitrogen (N)^18^.

### Cell line and treatment

**Fig. 1:**
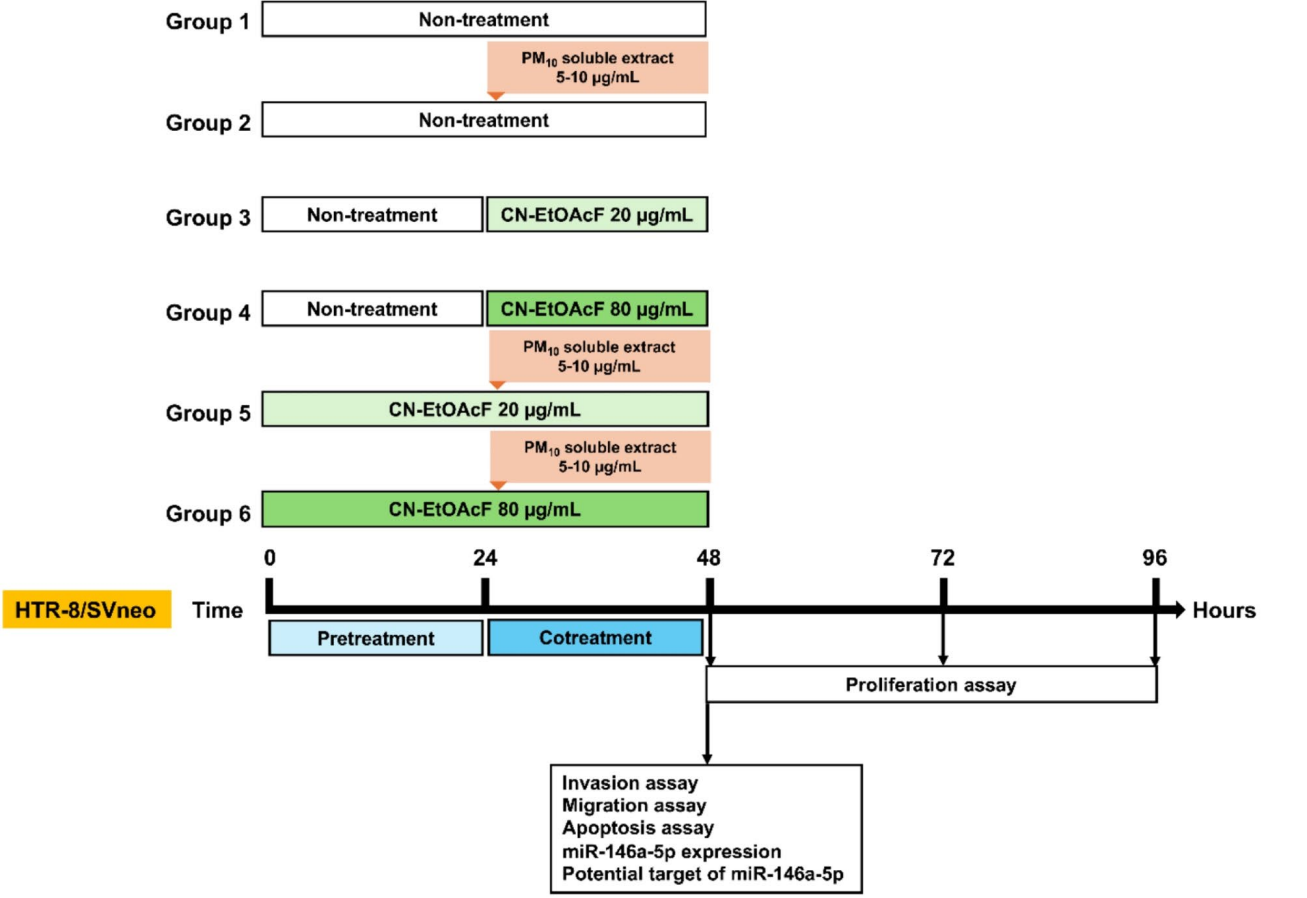
A schematic diagram of HTR-8/SVneo cell exposure schedule.

The trophoblast cell line, HTR-8/SVneo (RRID: CVCL_7162), was generously provided by Prof. Charles H. Graham from Kingston, Canada. These cells were grown in RPMI medium (Thermo Fisher Scientific, Dreieich, Germany), supplemented with 1% penicillin/streptomycin (Thermo Fisher Scientific, Dreieich, Germany) and 10% heat-activated FBS (Sigma-Aldrich, Darmstadt, Germany) at 37 °C with 5% CO_2_. Cells were subjected to different treatments and divided into six groups: control, 5 µg/mL PM_10_ soluble extract, 20 µg/mL CN-EtOAcF, 80 µg/mL CN-EtOAcF, pre-treatment for 24 h with 20 µg/mL CN-EtOAcF followed by co-treatment for 24 h with PM_10_ soluble extract, and pre-treatment for 24 h with 80 µg/mL CN-EtOAcF followed by co-treatment for 24 h with PM_10_ soluble extract (Fig. 1). After incubation, cells were harvested and analyzed for cell proliferation at 24, 48, and 72 h, as well as for migration, invasion, apoptosis, miR-146a-5p expression, and its potential targets.

### Cytotoxicity assay

Cell viability was assessed using the 3-(4,5-dimethylthiazol-2-yl)-2,5-diphenyltetrazolium bromide (MTT) reduction assay (Sigma-Aldrich, Darmstadt, Germany). A density of 5 × 10^3^ HTR-8/SVneo cells/well was seeded in a 96-well plate. The cells were exposed to PM_10_ soluble extract or CNE (ethyl acetate fraction; CN-EtOAcF) for 24–72 h, prior to 4 h of MTT solution (100 µL) at 37 °C. The formazan crystals were dissolved using an equal volume of dimethyl sulfoxide (DMSO), and the absorbance at 570 nm was measured with a microplate reader.

### Matrigel invasion and transwell migration assay

Cell invasion and migration were evaluated using hanging cell culture inserts with a pore size of 8 μm, placed in a 24-well plate (Sigma-Aldrich, Darmstadt, Germany). HTR-8/SVneo cells (1 × 10^5^) were pretreated with CN-EtOAcF and then challenged with PM_10_ soluble extract in serum-free medium. These cells were subjected to Matrigel pre-coated membranes (Corning, AZ, USA) for the invasion experiment or, alternatively, Transwell uncoated inserts for the migration assay. To act as a chemoattractant, 20% FBS was added to medium in the insert’s lower compartment. The cells that invaded the lower part of the membrane were preserved with cold ethanol, followed by staining with crystal violet, and their absorbance was measured at 570 nm after being discolored with acetic acid.

### Proliferation assay

The cell proliferation ELISA, BrdU (Sigma-Aldrich, Darmstadt, Germany) was utilized to quantify cell proliferation. HTR-8/SVneo cells (5 × 10^3^ cells/well) were seeded in a 96-well plate and treated to CN-EtOAcF and PM_10_ soluble extract at various concentrations for 24–72 h. Following this exposure, BrdU-containing medium was introduced for an additional 2 h to allow for the incorporation of BrdU into the cells. The BrdU-incorporated cells were subsequently fixed and subjected to incubation with a monoclonal anti-BrdU antibody conjugated with peroxidase. After washing, the cells underwent incubation with a substrate and the process was completed with 1 M H_2_SO_4_. Absorbance was evaluated at 450/690 nm using a microplate reader.

### Intracellular ROS assay

To assess the inhibition of intracellular reactive oxygen species (ROS) linked to *C. nervosum* extract, we employed the fluorometric intracellular ROS kit (MAK143; Sigma, St. Louis, MO, USA) following the manufacturer’s instructions. Initially, cells (5 × 10^3^ cells/ well) were seeded in a black 96-well plate and maintained overnight. Subsequently, the cells were treated with CN-EtOAcF. Following a 24 h incubation, 100 µM H_2_O_2_ was added to induce oxidative stress for 1 h. Then, the Master Reaction Mix was introduced into the treated cells, and the intensity of the fluorescence was detected using a fluorescence microplate reader with an excitation/emission wavelength of 490/525 nm.

### Apoptosis assay

Apoptosis was evaluated utilizing the Annexin V-FITC Apoptosis Detection Kit (BD, New Jersey, USA) following the manufacturer’s instructions. Cells underwent pre-treatment with CN-EtOAcF for 24 h and, followed by co-treatment with PM_10_ soluble extract for an additional 24 h. Subsequently, cells (1 × 10^6^ cells/mL) were resuspended in 1X annexin-binding buffer. Annexin V and PI were added to cell suspensions and incubated for 15 min in the dark. Analysis of apoptotic cells was conducted using flow cytometry (BD FACSLyric™, Becton Dickinson, Waltham, MA, USA).

### RNA isolation

Total RNA was isolated from cells subjected to either pre-treatment with CN-EtOAcF and PM_10_ soluble extract co-treatment or from untreated cells utilizing Trizol reagent (Invitrogen, Darmstadt, Germany) in accordance with the manufacturer’s instructions. The concentrations of total RNA were quantified using a NanoDrop spectrophotometer (PeqLab Biotechnologies GmbH, Erlangen, Germany), and samples exhibiting an A260/A280 ratio exceeding 1.8 were preserved at −80 °C for future use.

### Quantification of miR146a-5p expression by qRT–PCR

The qRT–PCR experiment was carried out using QIAquant™ 96 (Qiagen, Düsseldorf, Germany) following reverse transcription into cDNA with the miRCURY LNA Reverse Transcription Kit (Düsseldorf, Germany) following the manufacturer’s protocol. Subsequently, miRCURY LNA SYBR Green PCR kit was performed for miRNA analysis. Expression of miR-146a-5p (YP0020/NR_002745) (Qiagen, Düsseldorf, Germany) was calculated using the 2^−ΔΔCt^ method, using SNORD48 SNORD48 (YP0020/NR_002745) (Qiagen, Düsseldorf, Germany) as a reference.

### Target of miRNA prediction

miRNA targets prediction was conducted using a miRNA target prediction database including TargetScan RRID: SCR_010845 (http://www.targetscan.org/vert_71/), PicTar RRID: SCR_003343 (https://pictar.mdc-berlin.de), and miRDB RRID: SCR_010848 (http://mirdb.org).

### Quantitative RT–PCR (qRT–PCR)

After converting RNA into cDNA using RevertAid RT Reverse Transcription Kit from Thermo Fisher Scientific, Waltham, MA, USA, the expression levels of IL-1β, IL-6, TNF-α, and SOX5 were analyzed with the Maxima SYBR Green qPCR Master Mix from Thermo Fisher Scientific, Waltham, MA, USA, using the QIAguant™ 96 system (Qiagen, Düsseldorf, Germany). Gene expression was quantified the 2^−ΔΔCt^ method and normalized to GAPDH as a reference. The primers used in this study are shown in Table 1.

**Table 1:** List of primers.

Primers	Forward (5ʹ-3ʹ)	Reverse (5ʹ-3ʹ)
IL-1β	GCACAGTTCCCCAACTGGTA	AAGACACGGGTTCCATGGTG
IL-6	AGACAGCCACTCACCTCTTCAG	TTCTGCCAGTGCCTCTTTGCTG
TNF-α	CCCAGGCAGTCAGATCATCTTC	AGCTGCCCCTCAGCTTGA
SOX5	CAGCCAGAGTTAGCACAATAGG	CTGTTGTTCCCGTCGGAGTT
GAPDH	AGCCACATCGCTCAGACAC	GCCCAATACGACCAAATCC

### Statistical analysis

Data from three different experiments were presented as mean ± SD. Statistical analysis was performed using one-way analysis of variance (ANOVA), with significance determined at a *P*-value < 0.05. All statistical assessments were carried out using GraphPad Prism version 8.0, RRID: SCR_002798 (GraphPad Software, CA, USA).

## Results

### Extraction yield and phytochemical determination

The percentage of the yield for the crude extract was calculated relative to the weight of dried *C. nervosum*, while the yield for the partitioned fraction was determined based on the weight of the initial crude ethanolic extract used^38^. In this study, we subjected 200 g of *C. nervosum* to a 24 h extraction process using 70% ethanol, resulting in the production of an ethanolic crude extract (ECE) with a yield of 31.5%. The ECE was subsequently subjected to liquid-liquid partitioning using hexane (CN-HEXF), dichloromethane (CN-DCMF), ethyl acetate (CN-EtOAcF), and followed by a water (CN-WTF) extraction, resulting in yields of 0.64%, 0.27%, 3.73%, and 86.51%, respectively (Table 2). Notably, CN-WTF exhibited the highest yield among the partitioned fractions.

**Table 2:** Extraction and partitioning yields of CNE.

Fraction	Weight (g)	% Yield
Ethanolic crude extract (ECE)	63.0	31.5
Hexane fraction (CN-HEXF)	0.40	0.6
Dichloromethane fraction (CN-DCMF)	0.17	0.3
Ethyl acetate fraction (CN-EtOAcF)	2.35	3.7
Water fraction (CN-WTF)	54.50	86.5

**Fig. 2:**
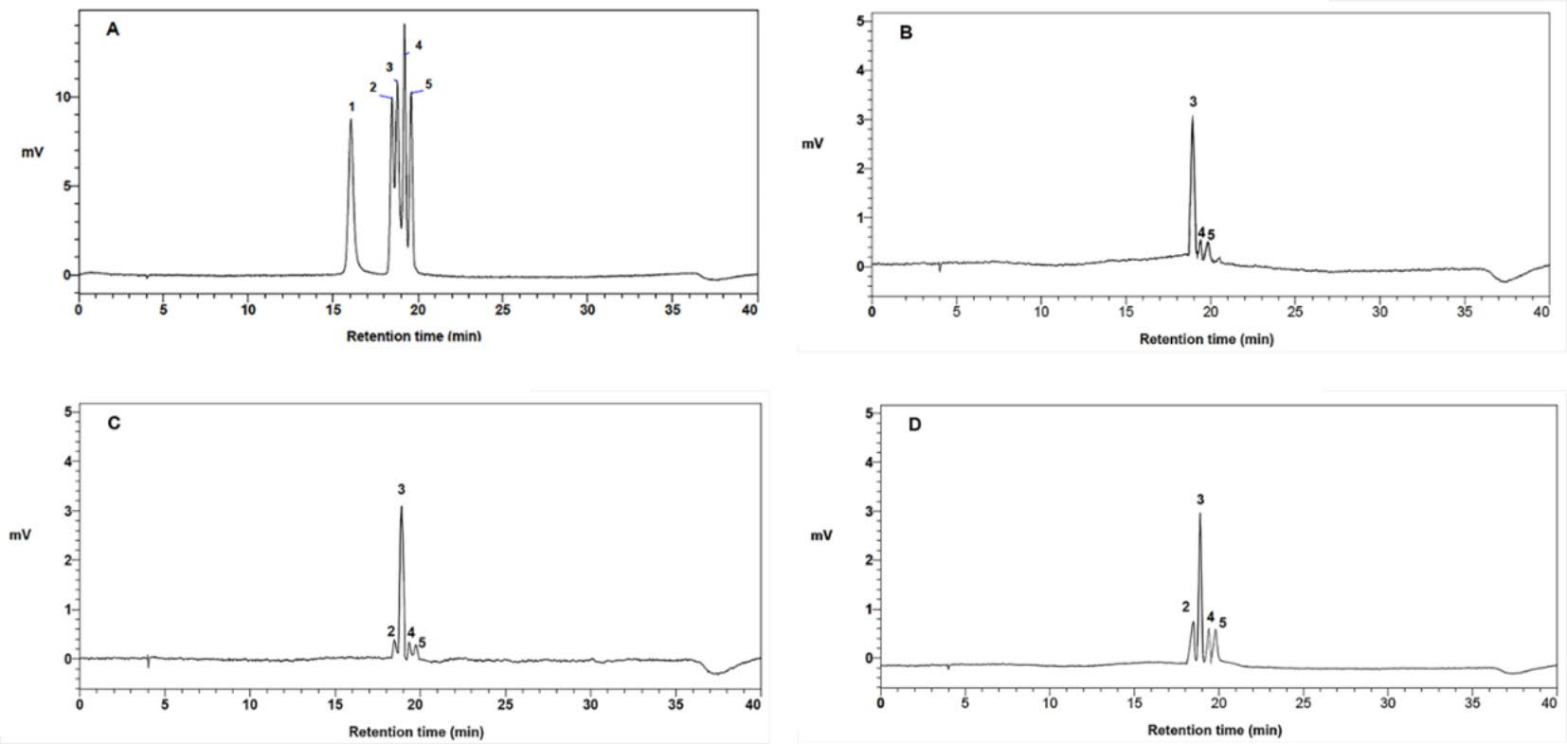
HPLC chromatograms of standard anthocyanins (**A**) and analyzed samples of ECE (**B**), CN-EtOAcF (**C**) and CN-WTF (**D**). Peak 1 represents Cyanidin-3,5-diglucoside, Peak 2 represents Cyanidin-3-O-glucoside chloride, Peak 3 represents Cyanidin-3-O-rutinoside chloride, Peak 4 represents Pelargonidin-3-O-glucoside chloride and Peak 5 represents Peonidin-3-O-glucoside chloride.

Next, we conducted an analysis of the total phenolic content (TPC), total flavonoid content (TFC), and anthocyanin content in the CNE. Our results indicated that CN-EtOAcF had the highest TPC (53.1 ± 0.48 mg GAE/g extract), while the lowest TPC was found in CN-WTF (12.70 ± 0.1 mg GAE/g extract). CN-DCMF exhibited the highest total flavonoid content (3.1 ± 0.84 mg CE/g extract), and CN-EtOAcF and CN-WTF had the lowest TFC, with values of 0.05 ± 0.3 and 0.05 ± 0.2 mg CE/g extract, respectively. Additionally, as shown in Table 3, the highest amount of anthocyanins, 29.5 ± 3.22 mg/g extract, was found in ECE, whereas the lowest amount, 0.03 ± 0.1 mg/g extract, was observed in CN-HEXF. In the present study, five anthocyanin standards analyzed through HPLC at 520 nm revealed retention times (RT) including Cyanidin-3,5-diglucoside (Peak 1, RT = 16.043 min), Cyanidin-3-O-glucoside chloride (Peak 2, RT = 18.466 min), Cyanidin-3-O-rutinoside chloride (Peak 3, RT = 18.933, Pelargonidin-3-O-glucoside chloride (Peak 4, RT = 19.374), and Peonidin-3-O-glucoside chloride (Peak 5, RT = 19.821) (Fig. 2A). The identification and quantification of anthocyanins in the extracts were determined by measuring the retention times and area under the peaks obtained from HPLC. Three anthocyanins were detected in ECE (Fig. 2B), whereas CN-EtOAcF (Fig. 2C) and CN-WTF (Fig. 2D) contained four anthocyanidins. None of anthocyanin was detected in CN-HEXF and CN-DCMF. The main anthocyanin in CN-EtOAcF, ECE, and CN-WTF was Cyanidin-3-O-rutinoside chloride, with concentrations of 79.90, 80.58, and 85.34 mg/100 g extract, respectively (Table 4). Total anthocyanin content was lowest in ECE, with a value of 96.77 mg/100 g extract, while CN-EtOAcF had the highest total anthocyanin content at 121.11 mg/100 g extract. These results indicate that CN-EtOAcF demonstrated the highest total anthocyanin content, which is consistent with the levels of anthocyanins determined using the pH differential spectroscopic method.

**Table 3:** Total phenolics, total flavonoid and anthocyanins content.

Fraction	TPC (mg GAE/g extract)	TFC (mg CE/g extract)	Anthocyanins (mg/g extract)
Ethanolic crude extract (ECE)	18.70 ± 0.4	0.54 ± 0.3	29.52 ± 3.2
Hexane fraction (CN-HEXF)	32.87 ± 0.4	2.87 ± 1.4	0.03 ± 0.1
Dichloromethane fraction (CN-DCMF)	34.66 ± 0.7	3.06 ± 0.8	0.79 ± 0.1
Ethyl acetate fraction (CN-EtOAcF)	53.13 ± 0.5	0.05 ± 0.3	35.04 ± 0.4
Water fraction (CN-WTF)	12.70 ± 0.1	0.05 ± 0.2	24.37 ± 2.0

**Table 4:** Individual and total anthocyanin content in *C. Nervosum* extracts.

Anthocyanins	Retention time (min)	Amount of anthocyanins (mg/100 g)				
		ECE	CN-HEXF	CN-DCMF	CN-EtOAcF	CN-WTF
1. Cyanidin-3,5-diglucoside	16.043	nd	nd	nd	nd	nd
2. Cyanidin-3-O-glucoside chloride	18.466	nd	nd	nd	16.44	14.94
3. Cyanidin-3-O-rutinoside chloride	18.933	80.58	nd	nd	79.90	85.34
4. Pelargonidin-3-O-glucoside chloride	19.374	7.42	nd	nd	12.47	7.71
5. Peonidin-3-O-glucoside chloride	19.821	8.77	nd	nd	12.30	9.78
Total anthocyanins		96.77	–	–	121.11	117.77

*nd not detectable.

### Antioxidant capacity of *C. Nervosum* extracts

When evaluating the antioxidant capacity of CNE using a range of methods, including ABTS, DPPH, and FRAP, consistent results from all three methods indicated that CN-EtOAcF exhibited the highest radical scavenging activity, followed by CN-HEXF, CN-DCMF, ECE, and finally, CN-WTF, as indicated in Table 5. The IC_50_ value of ABTS assay ranged from 43.77±0.7 (CN-EtOAcF) to 302.45±11.2 µg/mL (CN-WTF). The DPPH assay showed that IC_50_ was lowest at 79.98±4.4 µg/mL (CN-EtOAcF) and highest at 710.44±15.1 µg/mL (CN-WTF). In FRAP assay, CN-WTF had the lowest value at 54.10±7.0 µM Fe^2+^/g, while the highest value, 564.18±9.3 µM Fe^2+^/g, was found in CN-EtOAcF.

**Table 5:** Antioxidant activities of CNE.

Fraction	ABTS IC_50_ (µg/mL)	DPPH IC_50_ (µg/mL)	FRAP µM Fe^2+^/g
Ethanolic crude extract (ECE)	223.26 ± 10.7	457.03 ± 7.8	68.85 ± 4.1
Hexane fraction (CN-HEXF)	83.83 ± 2.6	250.15 ± 35.1	98.07 ± 8.8
Dichloromethane fraction (CN-DCMF)	96.96 ± 6.1	402.66 ± 15.8	87.02 ± 7.9
Ethyl acetate fraction (CN-EtOAcF)	43.77 ± 0.7	79.98 ± 4.4	564.18 ± 9.3
Water fraction (CN-WTF)	302.45 ± 11.2	710.44 ± 15.1	54.10 ± 7.0

### Chemical characteristics and size distribution of PM_10_

**Fig. 3:**
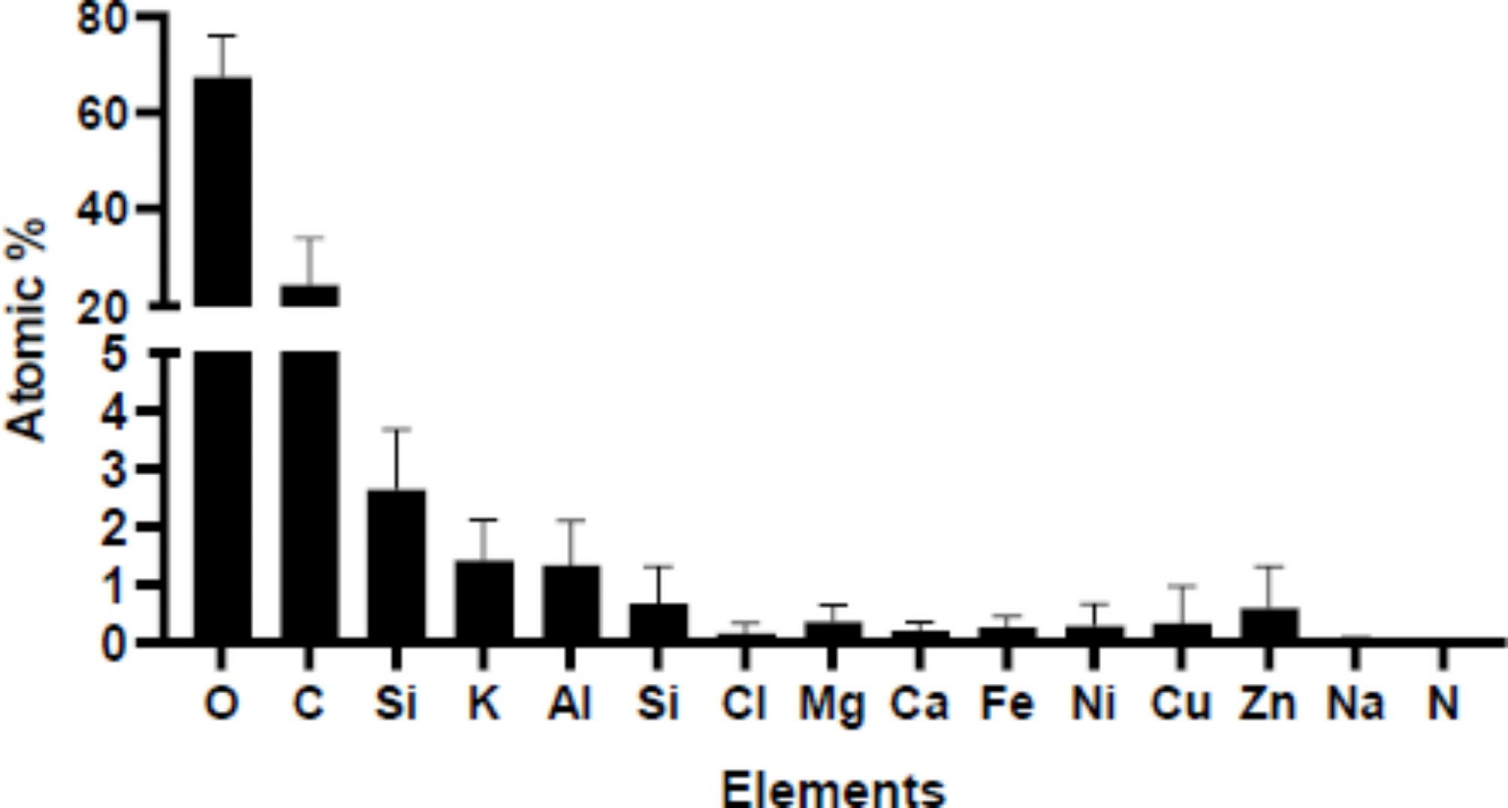
The elements components in PM_10_ samples expressed as atomic percentages.

**Fig. 4:**
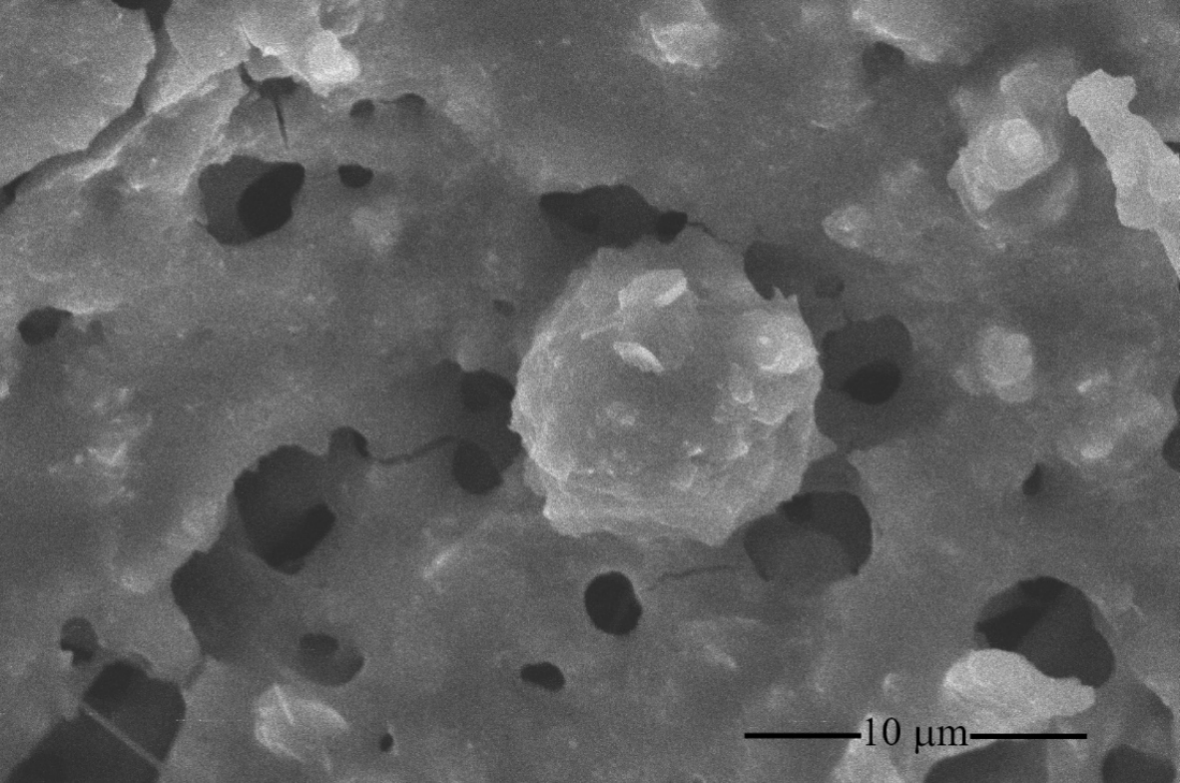
SEM image depicting PM_10_ particles at a ×12,000 magnification level.

To carry out elemental composition (O, C, Si, K, Al, S, Cl, Mg, Ca, Fe, Ni, Cu, Zn, Na, and N) of PM_10_, the filter was sectioned and subjected to SEM-EDX. The findings indicated that O was the predominant element (67%), followed by C (24%), Si (2.6%), K (1.4%), and Al (1.3%), as indicated in Fig. 3. The other elements were found less than 1%, whereas N was not detectable. Furthermore, the size of PM_10_ was determined to be below 10 μm, and it displayed a nearly spherical form, as illustrated in Fig. 4.

### Effect of PM_10_ soluble extract and CN-EtOAcF on trophoblast cell cytotoxicity

**Fig. 5:**
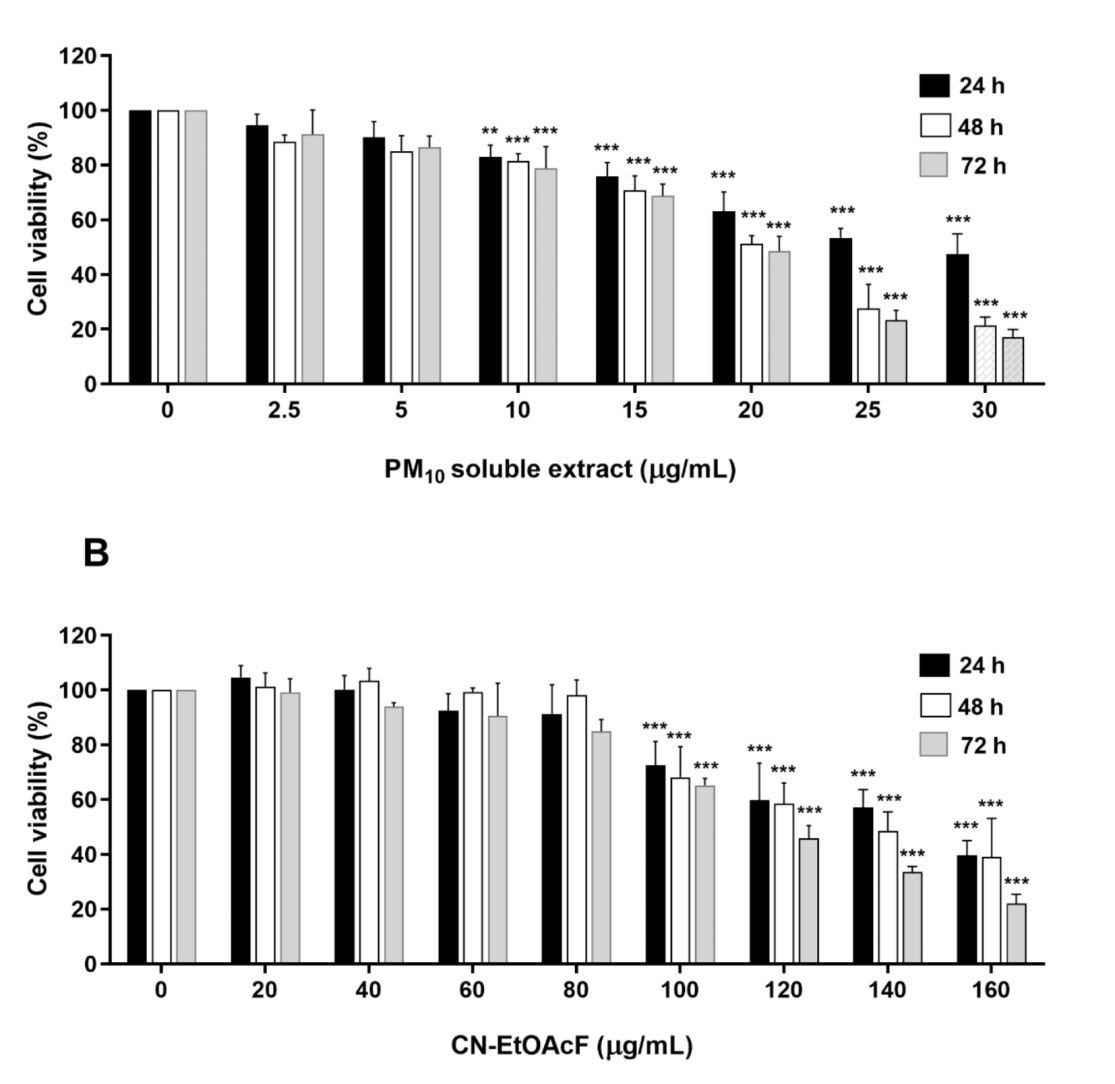
Cell viability of PM_10_ soluble extract (A) and CN-EtOAcF (B) on HTR-8/SVneo cells. MTT assay was conducted to evaluate PM_10_ soluble extract and CN-EtOAcF-treated cells for 24–72 h.

First, we assessed the cytotoxicity of PM_10_ soluble extract and CN-EtOAcF on HTR-8/SVneo cells using the MTT assay, and non-toxic doses were subsequently employed for further experiments. The results revealed that PM_10_ soluble extract significantly reduced HTR-8/SVneo cell viability at concentrations ranging from 10 to 30 µg/mL for 24–72 h (Fig. 5A). Furthermore, CN-EtOAcF, at concentrations of 100–160 µg/mL, exhibited toxic effects on HTR-8/SVneo cell viability over the same 24–72 h period (Fig. 5B).

### Effect of PM_10_ soluble extract and CN-EtOAcF on trophoblast migration and invasion

**Fig. 6:**
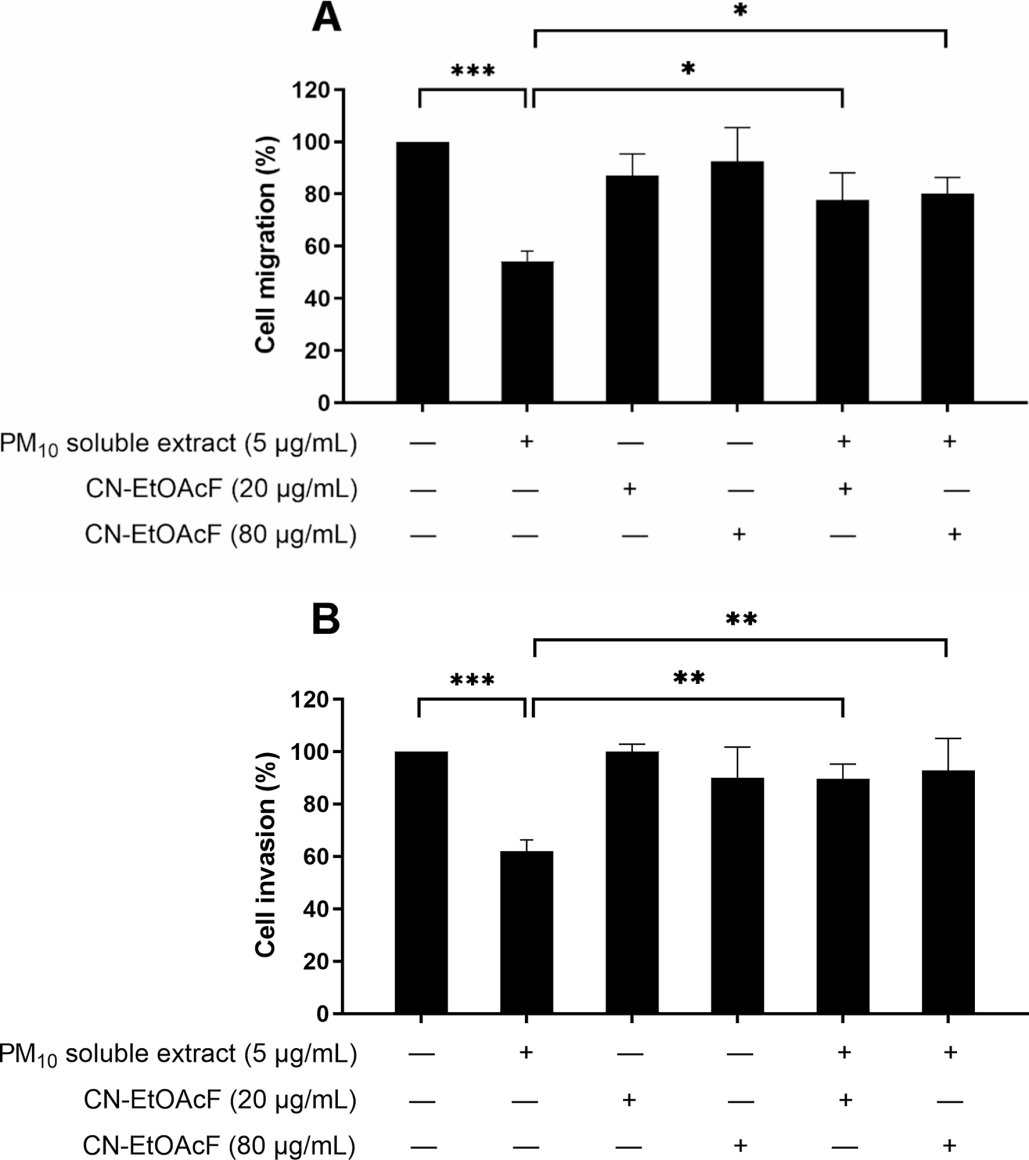
Cell migration (**A**) and invasion (**B**) of PM_10_ soluble extract and CN-EtOAcF on HTR-8/SVneo cells were assessed. Transwell migration and Matrigel invasion assays were conducted to evaluate the effect of CN-EtOAcF on PM_10_-induced cell migration and invasion for 24 h.

We analyzed the impact of CN-EtOAcF on trophoblast migration and invasion after PM_10_ soluble extract treatment. The outcomes revealed a notable increase in PM_10_ soluble extract-induced trophoblast migration when CN-EtOAcF was present, in comparison to PM_10_ soluble extract treatment alone (Fig. 6A). Likewise, at a concentration of 5 µg/mL, PM_10_ soluble extract significantly inhibited cell invasion. Trophoblast invasion was elevated in the pre-treatment of CN-EtOAcF and followed by PM_10_ co-treatment (Fig. 6B). These results suggest that CN-EtOAcF holds promise in preventing complications related to PM_10_ exposure during pregnancy.

### Effect of PM_10_ soluble extract and CN-EtOAcF on trophoblast cell proliferation

**Fig. 7:**
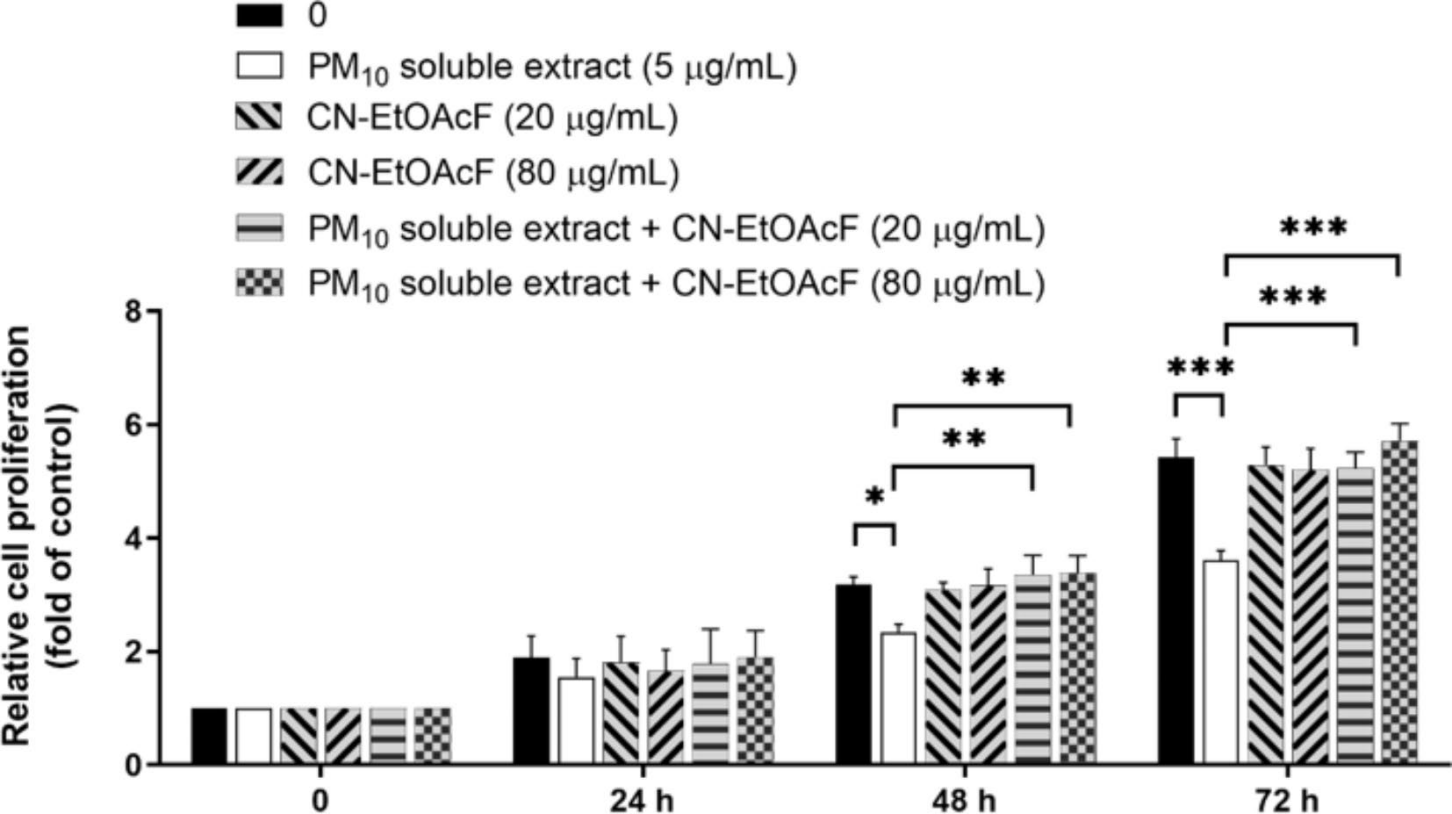
Cell proliferation of HTR-8/SVneo cells pre-treated with CN-EtOAcF and followed by PM_10_ soluble extract co-treatment was assessed. The BrdU assay was conducted to evaluate the effect of CN-EtOAcF on PM_10_ soluble extract-induced cell proliferation for 24–72 h.

To investigate the effect of PM_10_ soluble extract and CN-EtOAcF on trophoblast cell proliferation, a BrdU incorporation assay was performed over 24–72 h. Treatment of HTR-8/SVneo cells with 5 µg/mL PM_10_ soluble extract resulted in a suppression of cell proliferation specifically noticeable at 48–72 h (Fig. 7). Interestingly, pre-treatment of CN-EtOAcF at concentrations of 20 and 80 µg/mL followed by co-treatment of PM_10_ soluble extract demonstrated the ability to counteract the inhibitory effects of PM_10_ soluble extract on cell proliferation. These findings suggest that CN-EtOAcF could protect trophoblast cells from PM_10_ soluble extract-induced suppression of proliferation.

### Effect of CN-EtOAcF on intracellular ROS

**Fig. 8:**
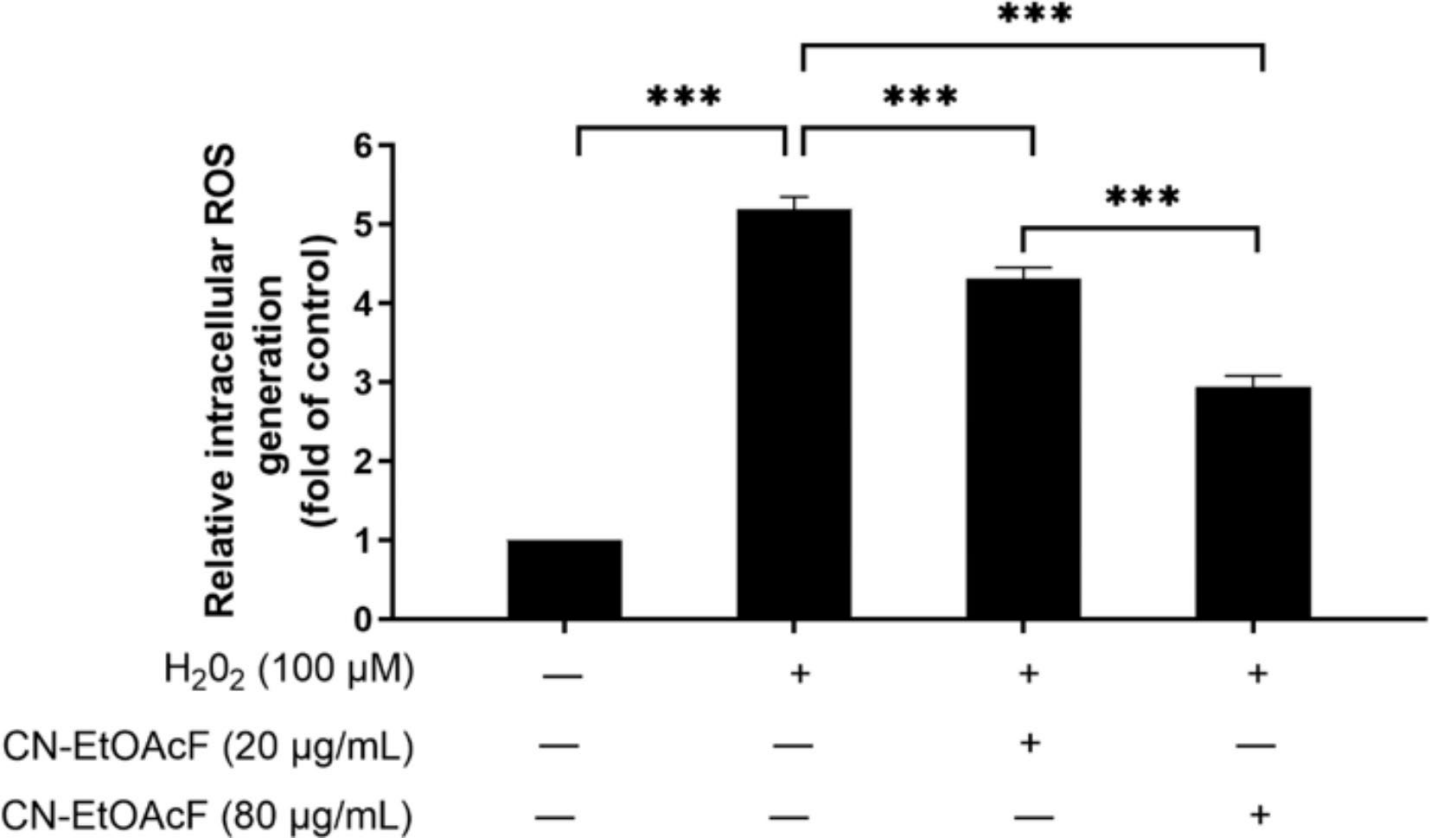
Intracellular ROS in HTR-8/SVneo cells. The florescence intensity was measured to evaluate the effect of CN-EtOAcF on H_2_O_2_-induced intracellular ROS.

The elevation of ROS is toxic and is associated with pollutants such as PM_10_, causing DNA damage. As shown in Fig. 8, we observed a significant dose-dependent reduction in intracellular ROS levels in CN-EtOAcF-pre-treated cells, followed by H_2_O_2_ co-treatment compared to H_2_O_2_-treated cells. Our findings suggest that CN-EtOAcF alleviates ROS production in trophoblast cells.

### Anti-inflammation by CN-EtOAcF

**Fig. 9:**
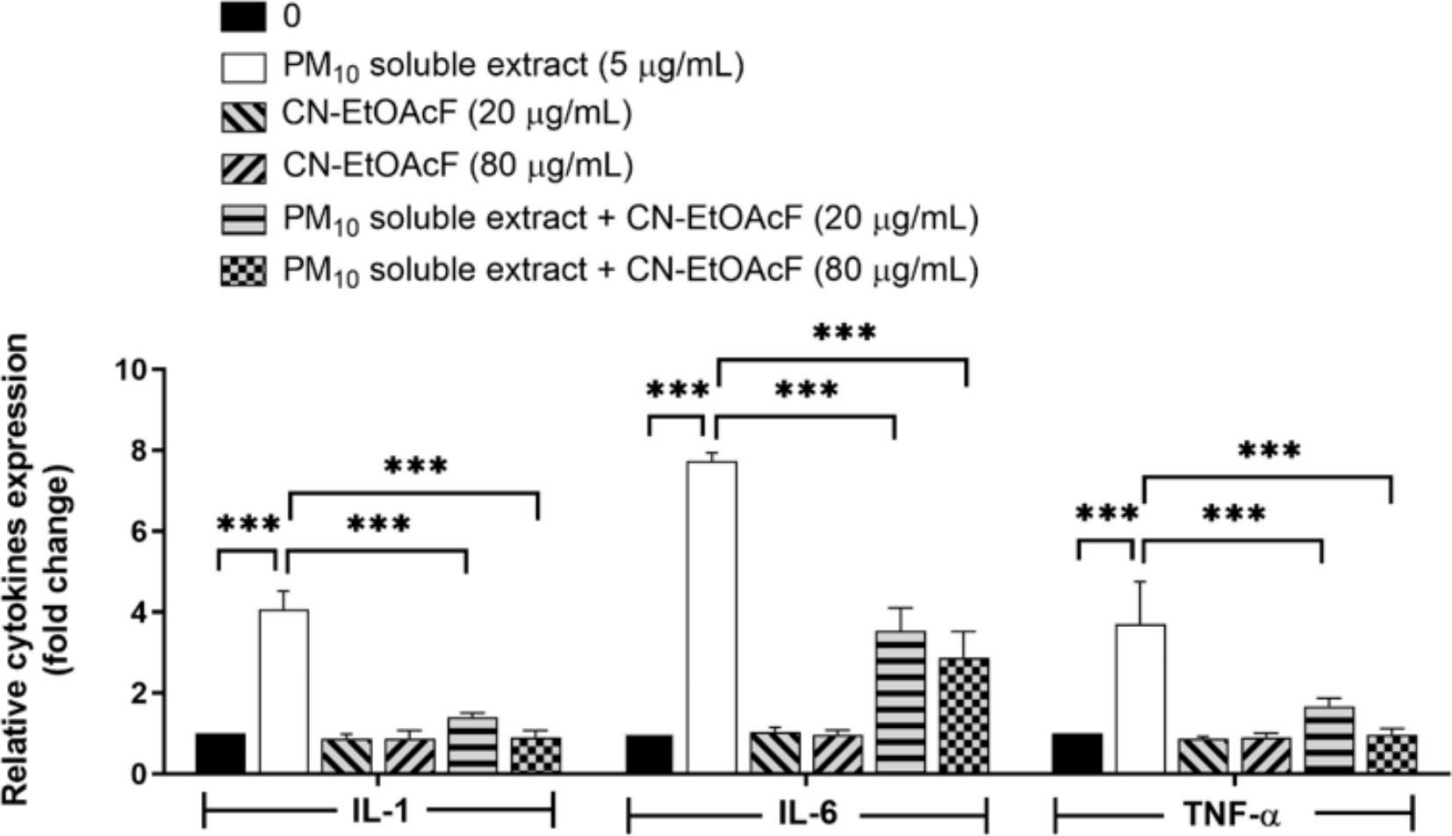
Cytokine expression in HTR-8/SVneo cells. Cells were pre-treated with CN-EtOAcF followed by co-treatment with PM_10_ soluble extract. RNA was isolated, and the expression levels of IL-1β, IL-6, and TNF-α were assessed using qRT-PCR.

To evaluate the anti-inflammatory properties of CN-EtOAcF on PM_10_ soluble extract-induced pro-inflammatory cytokine expression, we examined the levels of IL-1β, IL-6, and TNF-α in HTR-8/SVneo cells pre-treated with CN-EtOAcF and then co-treated with 5 µg/mL PM_10_ soluble extract (Fig. 9). Treatment with PM_10_ soluble extract alone led to a significant increase in the expression of IL-1β, IL-6, and TNF-α. Conversely, pre-treatment with CN-EtOAcF followed by co-treatment with PM_10_ soluble extract resulted in the suppression of these cytokines. These findings indicate that CN-EtOAcF exhibits anti-inflammatory activity in the presence of PM_10_ soluble extract.

### Effect of PM_10_ soluble extract and CN-EtOAcF on apoptosis

**Fig. 10:**
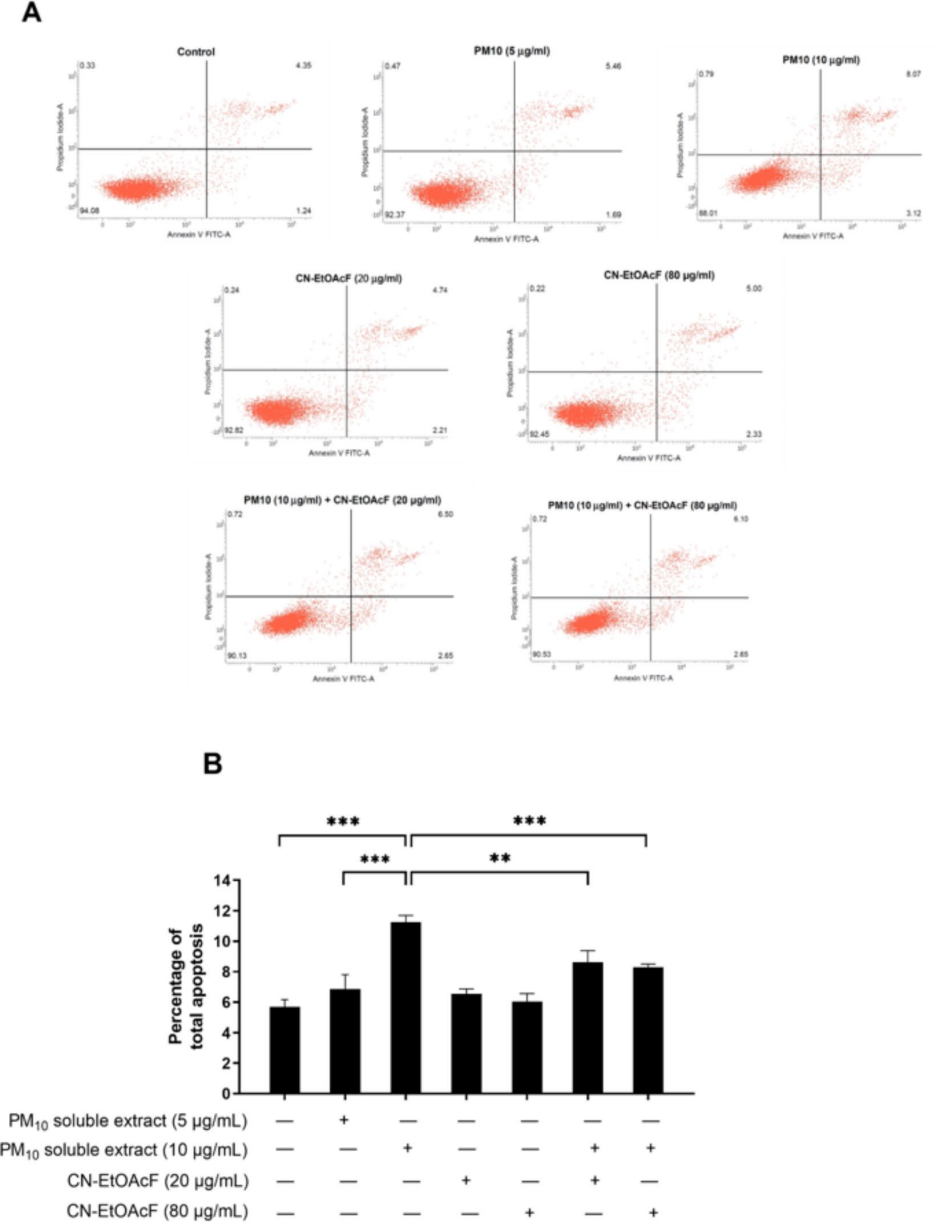
Apoptosis in HTR-8/SVneo cells. The Annexin V/PI apoptosis assay was performed on HTR-8/SVneo pretreated with CN-EtOAcF followed by PM_10_ soluble extract co-treatment, using flow cytometry (**A**). The histogram depicted the relative levels of apoptosis observed in each group (**B**).

Subsequently, we investigated the impact of CN-EtOAcF on apoptosis induced by PM_10_ soluble extract in trophoblast cells through Annexin V/PI staining using flow cytometry. Our findings reveal that apoptosis was induced in HTR-8/SVneo cells at a concentration of 10 µg/mL of PM_10_ soluble extract, with no significant effect observed at 5 µg/mL (Fig. 10A,B). Pre-treatment with CN-EtOAcF at concentrations of either 20 µg/mL or 80 µg/mL, followed by co-treatment with 10 µg/mL of PM_10_ soluble extract, effectively reversed apoptosis in HTR-8/SVneo cells. Thus, our results suggest that CN-EtOAcF has the potential to shield trophoblast cells from the detrimental effects of PM_10_ soluble extract by inhibiting cell apoptosis.

### Effect of CN-EtOAcF on PM_10_ soluble extract-induced miR-146a-5p expression and its potential target

**Fig. 11:**
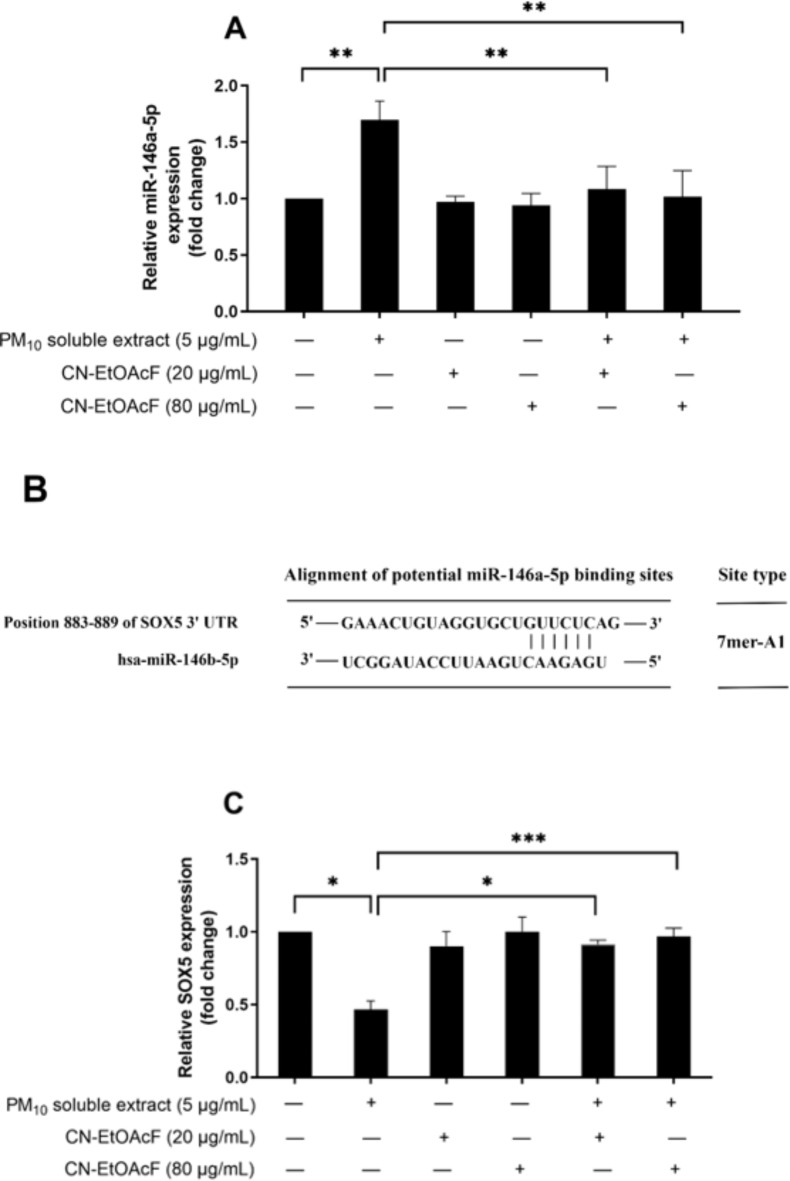
miR-146a-5p expression (**A**), the TargetScan database predicted the binding site of miR-146a-5p with SOX5 and SOX5 expression (**C**) in CN-EtOAcF-treated PM_10_ soluble extract-induced HTR-8/SVneo cells. Cells were pre-treated with CN-EtOAcF followed by PM_10_ soluble extract co-treatment for 24 h. The qRT-PCR was employed to quantify the expression of miR-146a-5p and SOX5.

Since CN-EtOAcF can restore growth inhibition, impaired migration and invasion, inflammation, and cell apoptosis caused by PM_10_ soluble extract in trophoblast cells, we hypothesize that pre-treatment of CN-EtOAcF could protect trophoblast cells from PM_10_ soluble extract co-treatment by modulating miR-146a-5p expression. Our results showed that following a 24 h treatment with 5 µg/mL PM_10_ soluble extract, there was an upregulation of miR-146a-5p in HTR-8/SVneo cells. Nevertheless, the administration of CN-EtOAcF at 20 and 80 µg/mL, subsequent to PM_10_ soluble extract co-treatment, led to a substantial downregulation of miR-146a-5p compared to PM_10_ soluble extract alone (Fig. 11A). We proceeded to predict the potential target of miR-146a-5p utilizing bioinformatics platforms. The finding indicated that 109 potential target genes of miR-146a-5p, including S0 × 5 were related to cell proliferation, migration and invasion (Supplementary Table 1). The results revealed that miR-146a-5p targeted a 7mer-A1 site located within the 3’ untranslated region (UTR) of SOX5, precisely at transcript positions 883–889 (Fig. 11B). Consequently, SOX5 was identified as a candidate target of miR-146a-5p, and its expression was validated through qRT-PCR authentication. The expression level of SOX5 was found to be opposite to that of miR-146a-5p (Fig. 11C). Our results suggest that CN-EtOAcF modulates the expression of miR-146a-5p, targeting SOX5.

### Discussion and conclusion

The trophoblast cell plays a pivotal role in ensuring a successful pregnancy, and any dysfunction in trophoblast activities can lead to pregnancy complications. Environmental pollutants such as PM_2.5_/_10_ trigger oxidative stress and inflammation, leading to the suppression of trophoblast cell growth, migration, invasion, and induction of cell cycle arrest and apoptosis^2,17,18^. PM_10_, predominantly composed of carbon and oxygen and extracted within a range of less than 10 μm, was identified as containing substances toxic to trophoblast cells. This discovery aligns with our prior findings^18^, suggesting that PM_10_ inhibits trophoblast cell proliferation, migration, invasion, and induces apoptosis. These factors contribute to adverse pregnancy outcomes, including hypertensive disorders, preeclampsia, preterm birth, low birth weight, and gestational diabetes mellitus^39,40^.

*C. nervosum* fruit extract (CNE) has been reported to possess various beneficial properties, including antidiabetic, neuroprotective, antiaging, antioxidant, and anti-inflammatory effects^32,41–43^. However, there is no existing report on the effectiveness of CNE in trophoblast cells. This study investigates and reports the protective effects of CNE on stress-inducing agent (PM_10_ soluble extract)-induced trophoblast cell dysfunction through the modulation of miR-146a-5p expression. Recently, miRNAs have emerged as crucial regulators of cell processes and homeostasis, operating at the post-transcriptional level through mRNA degradation or translational inhibition. Several miRNAs are associated with trophoblast functions, and altered miRNA expression leading to trophoblast cell dysfunction contributes to pregnancy complications^44^. One such miRNA is miR-146a-5p, known as a tumor suppressor in various cancers, capable of inhibiting cancer cell proliferation, migration, and invasion^27^. It also acts as an inflammatory miRNA, involved in regulating the NF-κB and NLRP pathways^28^. miR-146a-5p can be upregulated through stimulation by TNF-α, IL-1β, and LPS^45^, and its high expression level has been shown to decrease trophoblast cell proliferation, migration, and invasion^29^.

The correlation between TPC, anthocyanin levels, and radical scavenging activity is well-established in various fruits and vegetables^46–48^. High levels of TPC and anthocyanins contribute to robust antioxidant capacity, reducing intracellular ROS levels and inflammation^49^. In our study, we observed that TPC, anthocyanidin content, and antioxidant activity in CNE, particularly in *C. nervosum* fruit fractionated by ethyl acetate (CN-EtOAcF), correlate with its protective potential in trophoblast cells exposed to PM_10_ soluble extract. This protective effect is evident from the reversal of PM_10_ soluble extract-induced impairments in cell proliferation, migration, invasion, and apoptosis. CN-EtOAcF’s ability to mitigate ROS production and inflammatory cytokine expression further supports its role in protecting trophoblast cells from environmental stressors. It is well known that excessive intracellular ROS can damage proteins, nucleic acids, lipids, membranes, and organelles, potentially leading to the activation of apoptosis^50^. Our investigation revealed that pre-treatment with CN-EtOAcF, which exhibited the highest concentrations of TPC, antioxidant activities, and anthocyanins compared to other fractions, demonstrated a protective effect against PM_10_ soluble extract-induced cell damage by reducing intracellular ROS levels and reversing apoptosis in HTR-8/SVneo cells.

Additionally, CN-EtOAcF treatment led to decreased levels of inflammatory cytokines IL-1β, IL-6, and TNF-α in PM_10_-induced HTR-8/SVneo cells, which was associated with reduced expression of the inflammatory miRNA, miR-146a-5p. Pre-treatment with CN-EtOAcF prior to PM_10_ soluble extract exposure downregulated miR-146a-5p expression. This miRNA, whose gene is located on chromosome 10 (10q24.32), is known to regulate inflammation by targeting upstream of NF-κB signaling pathways^51–53^. Using the TargetScan database, we identified 109 potential target genes of miR-146a-5p involved in key cellular processes such as proliferation, migration, and invasion. Among these, SOX5 emerged as a notable target, linked to cell proliferation, migration, and invasion^27,54^. The expression pattern of SOX5 was inversely related to miR-146a-5p, with high levels of SOX5 regulating these processes. Our results suggest that miR-146a-5p influences trophoblast cell functions through a network of target genes, providing insights into its regulatory mechanisms in both physiological and pathological conditions. Further research, including gain- and loss-of-function studies, is needed to elucidate specific role of SOX5 and validate other identified target genes to assess the potential of miR-146a-5p as a therapeutic target for pregnancy-related complications.

Oxidative stress, characterized by an overproduction of ROS, which can be activated by PM_10_, leads to cellular damage, including DNA damage^55,56^. This damage contributes to cellular dysfunction, such as impaired cell proliferation, migration, invasion, and increased apoptosis^18,57,58^. The study revealed that PM_10_ soluble extract increased oxidative stress, suppressed trophoblast cell proliferation, migration, and invasion, and induced apoptosis. Remarkably, pre-treatment with CN-EtOAcF reduced oxidative stress, enhanced cell proliferation, migration, and invasion, and decreased apoptosis in trophoblast cells exposed to PM_10_ soluble extract. Several studies have demonstrated that CNE provides cellular protection through its antioxidant properties, anti-inflammatory effects, and its ability to prevent apoptosis across various cell types^32,59,60^. Consequently, our study highlights that CN-EtOAcF’s ability to reduce ROS levels, modulate inflammatory responses, and prevent apoptosis underscores its protective role against oxidative stress induced by PM_10_. Considering the essential role of trophoblast cells in placenta function during implantation and throughout pregnancy, enhancing their functionality including proliferation, migration, invasion while reducing apoptosis through pre-treatment with CN-EtOAcF, followed by co-treatment with the PM_10_ soluble extract, can mitigate the adverse effects of PM_10_ soluble extract. This approach could therefore contribute to a successful pregnancy by protecting trophoblast cells from oxidative stress and cellular damage induced by the environmental pollutant, PM_10_. It is indeed intriguing to elucidate whether the extract prevents PM_10_ from causing cellular damage through mechanisms such as precipitation, inactivation, and inhibition of cellular penetration and activation. Further investigation will be crucial for validating the proposed protective effects of the extract and understanding its potential therapeutic applications.

In summary, this study highlights the severe detrimental effects of PM_10_ soluble extract on trophoblast cells, which manifest through oxidative stress, particularly upregulation of miR-146a-5p, and subsequent disruptions in gene expression. These molecular changes impair key cellular processes such as proliferation, migration, and invasion, ultimately increasing apoptosis and compromising trophoblast cell function. However, the study demonstrates that pre-treatment with CN-EtOAcF effectively mitigates these harmful effects. By reducing oxidative stress, lowering ROS levels, and downregulating miR-146a-5p expression, CN-EtOAcF reverses the PM_10_ soluble extract-induced suppression of trophoblast cell proliferation, migration, invasion, and apoptosis. These findings underscore the protective potential of CN-EtOAcF in safeguarding trophoblast cells from PM_10_ soluble extract-induced damage, offering a promising therapeutic approach for maintaining normal cellular functions and supporting successful pregnancies in environments burdened by pollutants. Further investigations are crucial to fully elucidate the molecular mechanisms involved and to explore the broader therapeutic applications of CN-EtOAcF in counteracting environmental pollutant-induced cellular damage.

## Electronic supplementary material

Below is the link to the electronic supplementary material.

Supplementary Material 1

## Data Availability

The data produced by the present study can be accessed upon request from the corresponding author.
