# Supplementary material for: Cleistocalyx nervosum var. paniala mitigates oxidative stress and inflammation induced by PM10 soluble extract in trophoblast cells via miR-146a-5p
Source: Sci Rep. 2024 Oct 16;14:24265. doi: 10.1038/s41598-024-73000-y (PMC11484928; doi:10.1038/s41598-024-73000-y)
Supplement: Supplementary file 1 — Supplementary Material 1 [file 41598_2024_73000_MOESM1_ESM.pdf]

***Cleistocalyx nervosum* var *paniala* Mitigates Oxidative Stress and Inflammation Induced by PM_{10} Soluble Extract in Trophoblast Cells via miR-146a-5p**

Wittaya Chaiwangyen, Orawan Khantamat, Komsak Pintha, Napapan Kangwan, Amnard Onsa-ard,

Piyawan Nuntaboon, Angkana Songkrao, Pilaiporn Thippraphan, Dana Chaiyasit, Francisco Lázaro Pereira de Sousa

Supplementary Table 1. Target genes of miR-146-5p associated with cell proliferation, migration and invasion

No.	Target gene	Gene full name
1	IGSF1	immunoglobulin superfamily, member 1
2	IRAK1	interleukin-1 receptor-associated kinase 1
3	NOVA1	neuro-oncological ventral antigen 1
4	TRAF6	TNF receptor-associated factor 6, E3 ubiquitin protein ligase
5	NUMB	numb homolog (Drosophila)
6	NRAS	neuroblastoma RAS viral (v-ras) oncogene homolog
7	EIF4G2	eukaryotic translation initiation factor 4 gamma, 2
8	USP3	ubiquitin specific peptidase 3
9	AFAP1L2	actin filament associated protein 1-like 2
10	WWC2	WW and C2 domain containing 2
11	FBXO28	F-box protein 28
12	CARD10	caspase recruitment domain family, member 10
13	KLF7	Kruppel-like factor 7 (ubiquitous)
14	SCN3B	sodium channel, voltage-gated, type III, beta subunit
15	DNPEP	aspartyl aminopeptidase
16	PTPRA	protein tyrosine phosphatase, receptor type, A
17	FBXW2	F-box and WD repeat domain containing 2
18	NF2	neurofibromin 2 (merlin)
19	ZNF148	zinc finger protein 148
20	GDNF	glial cell derived neurotrophic factor
21	SMAD4	SMAD family member 4
22	APPL1	adaptor protein, phosphotyrosine interaction, PH domain and leucine zipper containing 1
23	ZNF652	zinc finger protein 652
24	MARCH6	membrane-associated ring finger (C3HC4) 6, E3 ubiquitin protein ligase
25	SIAH2	siah E3 ubiquitin protein ligase 2
26	RCAN1	regulator of calcineurin 1
27	PGK1	phosphoglycerate kinase 1

28	SORT1	sortilin 1
29	STC1	stanniocalcin 1
30	ACKR2	atypical chemokine receptor 2
31	ZNRF3	zinc and ring finger 3
32	MMP16	matrix metallopeptidase 16 (membrane-inserted)
33	SLC2A3	solute carrier family 2 (facilitated glucose transporter), member 3
34	POU3F2	POU class 3 homeobox 2
35	CELF3	CUGBP, Elav-like family member 3
36	GALNT10	UDP-N-acetyl-alpha-D-galactosamine:polypeptide N-acetylgalactosaminyltransferase 10 (GalNAc-T10)
37	CRB3	crumbs homolog 3 (Drosophila)
38	SYT1	synaptotagmin I
39	PIK3CB	phosphatidylinositol-4,5-bisphosphate 3-kinase, catalytic subunit beta
40	UBE2W	ubiquitin-conjugating enzyme E2W (putative)
41	CLCN6	chloride channel, voltage-sensitive 6
42	BTG2	BTG family, member 2
43	ZNF367	zinc finger protein 367
44	LCP2	lymphocyte cytosolic protein 2 (SH2 domain containing leukocyte protein of 76kDa)
45	RPA3	replication protein A3, 14kDa
46	PRKAA2	protein kinase, AMP-activated, alpha 2 catalytic subunit
47	SLC39A1	solute carrier family 39 (zinc transporter), member 1
48	NRP2	neuropilin 2
49	CDS1	CDP-diacylglycerol synthase (phosphatidate cytidylyltransferase) 1
50	FAM83F	family with sequence similarity 83, member F
51	FLOT2	flotillin 2
52	EHF	ets homologous factor
53	ROBO1	roundabout, axon guidance receptor, homolog 1 (Drosophila)
54	KIF26B	kinesin family member 26B
55	SMARCA5	SWI/SNF related, matrix associated, actin dependent regulator of chromatin, subfamily a, member 5
56	KCTD15	potassium channel tetramerization domain containing 15
57	RUNX1T1	runt-related transcription factor 1; translocated to, 1 (cyclin D-related)
58	MED1	mediator complex subunit 1
59	PRCP	prolylcarboxypeptidase (angiotensinase C)
60	SYT13	synaptotagmin XIII
61	WASF3	WAS protein family, member 3
62	KDM2B	lysine (K)-specific demethylase 2B
63	DYNLL2	dynein, light chain, LC8-type 2

64	BRD4	bromodomain containing 4
65	DLGAP2	discs, large (Drosophila) homolog-associated protein 2
66	UNC5D	unc-5 homolog D (C. elegans)
67	IMPA2	inositol(myo)-1(or 4)-monophosphatase 2
68	RNF32	ring finger protein 32
69	JAZF1	JAZF zinc finger 1
70	ADAM19	ADAM metallopeptidase domain 19
71	SP8	Sp8 transcription factor
72	BMPR1A	bone morphogenetic protein receptor, type IA
73	CAMSAP1	calmodulin regulated spectrin-associated protein 1
74	DUSP16	dual specificity phosphatase 16
75	MAN1C1	mannosidase, alpha, class 1C, member 1
76	EDNRB	endothelin receptor type B
77	ERBB4	v-erb-b2 avian erythroblastic leukemia viral oncogene homolog 4
78	WASF2	WAS protein family, member 2
79	NUCKS1	nuclear casein kinase and cyclin-dependent kinase substrate 1
80	USP44	ubiquitin specific peptidase 44
81	GRSF1	G-rich RNA sequence binding factor 1
82	NAIF1	nuclear apoptosis inducing factor 1
83	TAF9B	TAF9B RNA polymerase II, TATA box binding protein (TBP)-associated factor, 31kDa
84	LIN28A	lin-28 homolog A (C. elegans)
85	MYO6	myosin VI
86	MAPT	microtubule-associated protein tau
87	TMEM33	transmembrane protein 33
88	CELF1	CUGBP, Elav-like family member 1
89	ARMC8	armadillo repeat containing 8
90	PTGFRN	prostaglandin F2 receptor inhibitor
91	KCND3	potassium voltage-gated channel, Shal-related subfamily, member 3
92	KMT2D	lysine (K)-specific methyltransferase 2D
93	TCF21	transcription factor 21
94	SEMA3G	sema domain, immunoglobulin domain (Ig), short basic domain, secreted, (semaphorin) 3G
95	NSD1	nuclear receptor binding SET domain protein 1
96	KCMF1	potassium channel modulatory factor 1
97	IGF2R	insulin-like growth factor 2 receptor
98	PER1	period circadian clock 1
99	SOX5	SRY (sex determining region Y)-box 5
100	PBX2	pre-B-cell leukemia homeobox 2

101	UBA6	ubiquitin-like modifier activating enzyme 6
102	MTDH	metadherin
103	STAU2	staufen double-stranded RNA binding protein 2
104	RRAGD	Ras-related GTP binding D
105	ATG7	autophagy related 7
106	GRK5	G protein-coupled receptor kinase 5
107	GATA6	GATA binding protein 6
108	CD3D	CD3d molecule, delta (CD3-TCR complex)
109	PDHB	pyruvate dehydrogenase (lipoamide) beta
